# Underwater cricopharyngeal peroral endoscopic myotomy

**DOI:** 10.1055/a-2346-4794

**Published:** 2024-07-08

**Authors:** Pavithra Ramakrishnan, Nabeel Azeem

**Affiliations:** 1 14400 Internal Medicine, University of Minnesota Medical Center, Minneapolis, United States; 2 14400 Gastroenterology and Hepatology, University of Minnesota Medical Center, Minneapolis, United States

Peroral endoscopic myotomy (POEM) is a well-established third-space endoscopic technique that can be technically challenging when used to treat a cricopharyngeal bar, even for experienced operators, due to the limited working space in the hypopharynx and upper esophageal sphincter 1 2 . Here we propose the use of saline immersion to improve the technical success rate of the procedure.

We present the case of a 58-year-old woman with a history of inclusion body myositis for 10 years and dysphagia due to a symptomatic cricopharyngeal bar for 3 years ( Fig. 1 ). We performed cricopharyngeal POEM under general anesthesia. Once the cricopharyngeal bar was identified, thermal markings were made at the entry point <1 cm proximally using a triangle-tip knife. A submucosal bleb was created using indigo carmine and saline, followed by a transverse mucosotomy. A short submucosal tunnel was created using spray coagulation. To gain entry into the tunnel, facilitate further dissection, and ultimately perform the myotomy, saline immersion was used to lift the mucosa away from the cricopharyngeal muscle ( Video 1 , Fig. 2 ). The myotomy was extended 2 cm beyond the cricopharyngeal muscle to ensure complete division. The resistance at the hypopharynx was eliminated. The mucosotomy was closed longitudinally using four hemoclips ( Fig. 3 ). After the dysphagia had resolved and the esophagogram showed no leaks, the patient was discharged on day 3. Barium swallow performed 15 days after the procedure showed resolution of the cricopharyngeal bar and complete passage of barium ( Fig. 4 ). At the 1 month follow-up, the patient reported resolution of her dysphagia.

**Fig. 1:**
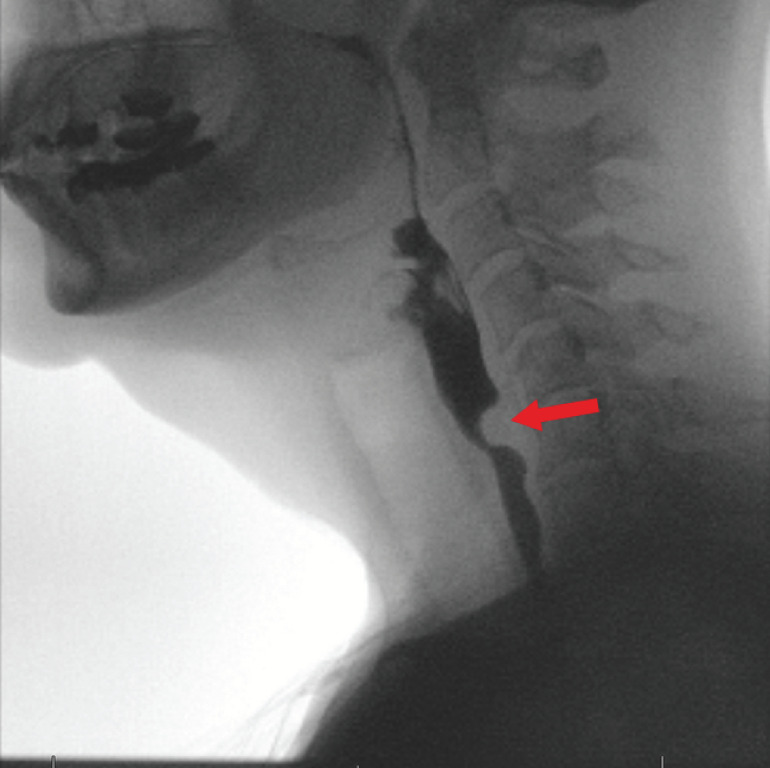
Barium swallow study showing a cricopharyngeal bar (red arrow) in a 58-year-old woman with dysphagia.

**Fig. 2:**
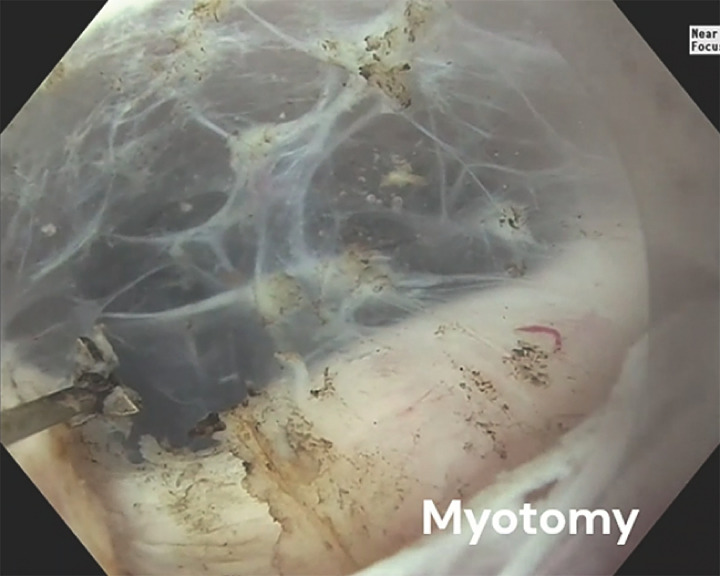
Endoscopic image of underwater tunnel and the uncut cricopharyngeal muscle in a 58-year-old woman with a cricopharyngeal bar.

**Fig. 3:**
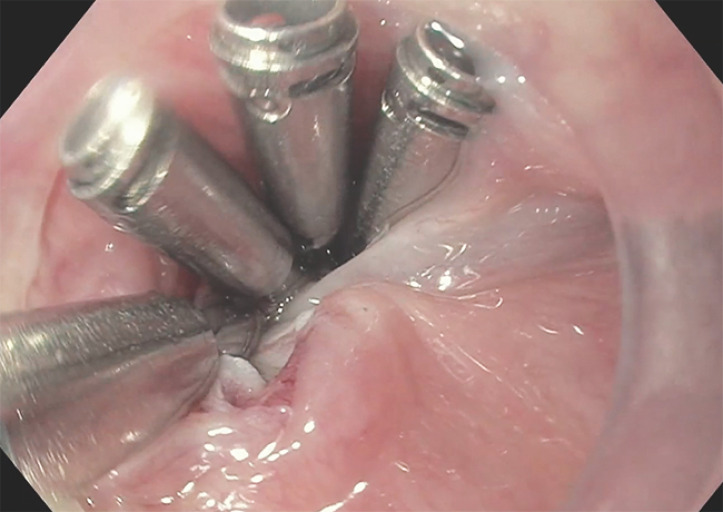
Four hemoclips were applied to repair the mucosotomy during cricopharyngeal peroral endoscopic myotomy.

**Fig. 4:**
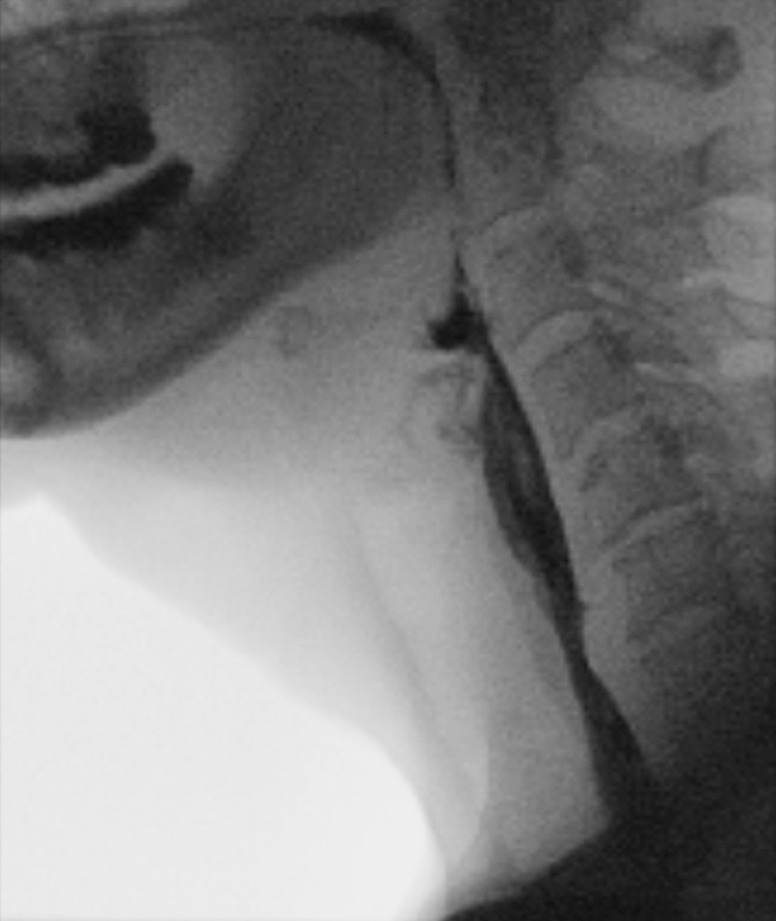
Barium swallow study performed after the cricopharyngeal peroral endoscopic myotomy showing resolution of the cricopharyngeal bar and complete passage of barium.

Application of the underwater technique to separate tissues during a successful cricopharyngeal per oral myotomy in a 58-year-old woman with a cricopharyngeal bar.Video 1

Underwater endoscopic mucosal resection, used for colorectal polyps as early as 2012, leverages the inherent density differences in tissue planes to facilitate tissue separation 3 . Given the technical difficulty of performing cricopharyngeal POEM, we believe that underwater endoscopic mucosal resection can enable easier entry into the tunnel and facilitate more complete myotomy.

Endoscopy_UCTN_Code_TTT_1AO_2AG_3AZ

## Notes

Endoscopy E-Videos https://eref.thieme.de/e-videos
*E-Videos* is an open access online section of the journal *Endoscopy* , reporting on interesting cases and new techniques in gastroenterological endoscopy. All papers include a high-quality video and are published with a Creative Commons CC-BY license. Endoscopy E-Videos qualify for HINARI discounts and waivers and eligibility is automatically checked during the submission process. We grant 100% waivers to articles whose corresponding authors are based in Group A countries and 50% waivers to those who are based in Group B countries as classified by Research4Life (see: https://www.research4life.org/access/eligibility/ ). This section has its own submission website at https://mc.manuscriptcentral.com/e-videos .
